# Associations of Feeding Practices in Early Life and Dietary Intake at School Age with Obesity in 10- to 12-Year-Old Arab Children

**DOI:** 10.3390/nu13062106

**Published:** 2021-06-19

**Authors:** Khitam Muhsen, Wasef Na’amnih, Rebecca Goldsmith, Maayan Maya, Nuha Zeidan, Eias Kassem, Asher Ornoy

**Affiliations:** 1 Department of Epidemiology and Preventive Medicine, School of Public Health, Sackler Faculty of Medicine, Tel Aviv University, Ramat Aviv, Tel Aviv 6997801, Israel; wasef25@yahoo.com (W.N.); maayanmaya@mail.tau.ac.il (M.M.); 2 Nutrition Division, Ministry of Health, Jerusalem 9101002, Israel; gorebecca@gmail.com; 3 Clalit Health Service, Diet and Nutrition Unit, P.O. Box 789, Arara 30026, Israel; nuhayz@gmail.com; 4 Department of Pediatrics, Hillel Yaffe Medical Center, Hadera 38100, Israel; EiasK@hy.health.gov.il; 5 Laboratory of Teratology, Department of Medical Neurobiology, The Hebrew University Hadassah Medical School, Jerusalem 9112002, Israel; 6 Adelson School of Medicine, Ariel University, Ariel 40700, Israel; asher.ornoy@mail.huji.ac.il

**Keywords:** early life exposures, infancy, school age, obesity, dietary intake

## Abstract

Understanding the role in pediatric obesity of early life feeding practices and dietary intake at school age is essential for early prevention. The study aimed to examine associations of early life feeding practices, environmental and health-related exposures, and dietary intake at school age as determinants of obesity in children aged 10–12 years. In an earlier study of 233 healthy infants in two Arab towns in northern Israel, neonatal history, feeding practices, and health information were obtained up to age 18 months. This follow-up study assessed dietary intake and anthropometric measurements at age 10–12 years using the 24 h recall method. Overall, 174 children participated in this study. Almost all (98%) the children were breastfed. The prevalence of obesity at school age was 42%. A multivariable model adjusted for energy intake and socioeconomic status showed positive associations of total fat intake and of weight-for-height z score, but not feeding practices in infancy, with obesity. Higher gestational age at birth was associated with lower odds of obesity at age 10–12 years. In conclusion, in a population with near universal breastfeeding, gestational age at birth, weight indicators but not feeding practices in infancy, and total fat intake at school age were associated with increased likelihood of obesity.

## 1. Introduction

Achieving optimal physical growth in children is essential to reduce health inequalities throughout the lifecycle. Early life and school age environmental and dietary exposures are the main determinants of children’s physical growth [1,2,3,4,5,6]. Birth weight and weight gain during early life were shown to be related to high body mass index (BMI) and obesity during childhood and adolescence [3,4,5,6], and to cardiovascular and metabolic risks in adulthood [7], as well as trajectories in BMI in childhood and obesity in adolescence [7,8,9]. Thus, understanding early determinants of obesity in children is warranted. 

Diet and feeding practices in infancy were shown to have long-term influence on children’s growth, including adiposity [10,11,12,13,14,15]. A study was undertaken in the Czech Republic of adolescents who were mostly breastfed during infancy (90.7%, based on parental recall); though only a small proportion (7.1%) was breastfed for more than six months [11]. An association was found between breastfeeding of any duration with a 20% reduction in the risk of overweight or obesity at age 14–16 years [11]. A prospective study from Western Australia concluded that a longer duration of breastfeeding might be protective against obesity at age 20 years [10]. Others, however, found no significant association between feeding practices in infancy and BMI [5].

Dietary intake at school age has also been shown to affect body composition and the risk of obesity [16]. For example, a higher protein intake at age eight years was associated with an increased risk of obesity/overweight and a higher fat-free mass (e.g., lean body mass, muscle) at age 10 years [16]. 

In light of the above-mentioned evidence, the role of dietary intake in early life and school age on children’s growth should be further explored. This is particularly important given the possible effects of cultural variation and socioeconomic inequalities that affect diet, children’s growth, and obesity risk. The primary objective of the current study was to examine associations of early life feeding practices, environmental and health-related exposures (such as birth weight and gestational age at birth), and childhood dietary intake with BMI and obesity at school age in Arab children in Israel. The Arab population, the main ethnic minority in Israel, is characterized by lower socioeconomic status (SES) and a higher burden of cardiovascular disease and diabetes compared to the Jewish population [17]. The assessment of childhood exposures that might affect BMI and obesity is necessary to identify at-risk groups and thus promote the design of better tailored preventive interventions to tackle childhood overweight and obesity. 

## 2. Materials and Methods

### 2.1. Study Population and Design 

The study was conducted in two Arab villages (referred to here as villages A and B) in northern Israel, located ~10 km apart, in the Hadera sub-district. The Arab population in Israel is in transition, marked by continuous improvement in educational level and in health indicators, such as increased life expectancy and declines in infant mortality and cardiovascular disease mortality. Despite this progress, disparities remain in SES and non-communicable diseases as compared to the Jewish population in Israel [17,18]. The Arab population is also characterized by a high proportion of consanguineous marriages, up to 31% [19,20]. This is associated with high rates of congenital anomalies, inherited diseases and infant mortality [20].

In 2017, 12,900 residents lived in village A and 14,400 in village B [21,22]. The mean years of education of adults aged 25–54 years was 10.2 in village A and 8.4 in village B, and both were lower than in the general population of Israel at 12.2. The respective average per capita monthly incomes in villages A and B were 2188 new Israeli Shekels (NIS) (~$633 USD) and 1944 NIS (~$562 USD), both lower than the national average of 3870 NIS (~$1119 USD) [22]. Given the differences between the two villages in SES indicators [22], we considered village B as lower SES and village A as higher SES. 

The traditional diet in the Arab population is based on the Mediterranean diet [23,24], including large portions of vegetables, fruit, fish and olive oil. However, shifts towards Westernized diets are evident, including manufactured food, sweets, and soft drinks [24,25,26]. Breastfeeding is common among Arab women; a national survey showed that during 2009–2012, about 87% of infants were breastfed at age two months [27].

The original cohort of the current study comprised 233 children: 134 (57.5%) from the lower SES village (B) and 99 (42.5%) from the higher SES village (A). The children were recruited at age 1–9 weeks, between January and August 2007, through the Maternal and Child Health clinics. The cohort has been described elsewhere [28,29]. Inclusion criteria were as follows: (1) a singleton birth; (2) no prenatal/peri-natal complications; (3) no birth defects or diseases that might affect growth; (4) birth weight >2 kg; and (5) gestational age at birth >34 weeks. Since this cohort was designed to address factors that could affect children’s growth and development, we included children (*n* = 223, 95.7%) born mainly at full term (at gestational age 37–42 weeks). However, to increase the sample size, we included 10 (4.3%) late preterm babies (gestational age at birth 34–36 weeks) who were overall healthy babies.

**Figure 1:**
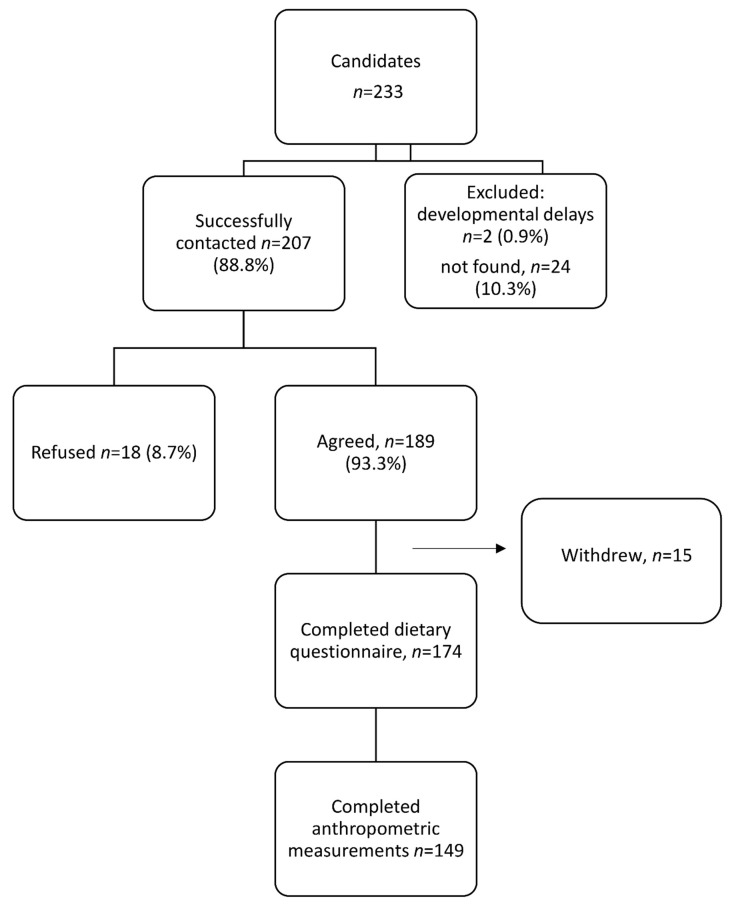
Flow chart of enrollment and data collection.

During 2017–2019, a follow-up examination was conducted of the same children, aged 10–12 years. Of the original 233 children in the cohort, one child died due to a road accident, two children with developmental delays and special needs were excluded from the current analysis, and 24 were not located (their families moved to other villages or they did not respond to our repeated attempts to contact). Overall, 207 (88.8%) families were successfully contacted. Of them, 18 (8.7%) refused to participate, 15 (7.2%) initially consented but subsequently withdrew their consent, and 174 (84.1%) agreed to participate in the study. Of the 174 children, 149 completed both the dietary intake and anthropometric measurements at age 10–12 years. Compliance to take part in the study among all candidate families was 75% (Figure 1) and was higher among families from village B than those from village A (82% vs. 65%), *p* = 0.002.

### 2.2. Data Collection and Definition of the Study Variables 

During 2007–2009, data on early life exposures were collected through maternal interviews at enrollment, follow-up interviews/visits and from medical records [28,29]. During 2017–2019, parents who provided informed consent were interviewed (face-to-face) in Arabic to collect updated socioeconomic data, children’s health status and dietary intake at school age.

### 2.3. Anthropometric Measurements

Height and weight were obtained by trained research assistants. Standing height (without shoes) was recorded to the nearest 0.1 cm. Weight was measured (with light clothing) to the nearest 0.1 kg using a calibrated digital scale. 

### 2.4. The Dependent Variables

Body mass index Z (BMIZ) score at school age (a continuous variable) was calculated using weight and height measurements compared to the World Health Organization (WHO) data for ages 5–19 years [30,31]. BMI was calculated as: weight (kg)/height^2^ (meters (m)). 

Obesity at school age (10–12 years) was defined according to the WHO classification as a BMIZ score >2 standard deviations (SD) compared to the WHO reference population (equivalent to BMI 30 kg/m^2^ at age 19 years). Children with BMIZ scores between ≥1SD and ≤2SD (equivalent to BMI between ≥25 and <30 kg/m^2^ at age 19 years) were classified as overweight. Children with BMIZ scores between ≥−2SD and <1SD were considered as having normal weight, while thinness was defined as BMIZ <−2SD [30].

### 2.5. Dietary Intake Questionnaire

Information was collected on dietary intake at age 10–12 years using the 24 h food-recall method. Twenty-four-hour dietary recall interviews (multiple pass) were conducted in-person in Arabic with the participants and their mothers, according to a protocol of the Israeli Ministry of Health (MOH) [24]. Using three passes, the interviewer asked the participant to report his/her food intake during the 24 h preceding the interview. The first pass was a quick list, in which the participants were asked to recall all that they had eaten and drunk during the 24 h prior to the interview. The second pass was a detailed description of all the items mentioned in the first pass (quick list). In the third pass, the interviewer assessed additional items of foods and beverages that might have been missed in the earlier passes. The Arabic version of the MOH “Food and Food Quantities Guide” was used to assist the participants in identifying foods and to quantify food during the dietary recall. The guide was designed to enhance the accuracy of dietary intake assessment and comprises pictures of common Israeli foods and comprehensive questions about food. The interviewers underwent standard training on performing these interviews. This included two lectures, exercises with supervised simulations, real-life exercises, and feedback. Quality control on the collected data was performed to assess completeness and coherence. Feedback was provided to the interviewers as needed. 

### 2.6. The Main Independent Variables 

Dietary intake at school age was classified based on the 24 h food-recall questionnaires as described above. Data from these questionnaires were entered to the MOH’s computerized dietary analysis program “TZAMERET” and database, which includes the Israeli national nutrient database. Reports of macronutrient and micronutrient intake were generated. Dietary intake of nutrients was computed as a continuous variable and as a categorical variable using tertiles.

Feeding practices in early life were defined based on maternal reports in interviews conducted at enrollment and at ages 2, 4, 6, 8, 12, and 18 months using a structured questionnaire. Information was collected on the infants’ feeding habits: breastfeeding (yes or no), duration of breastfeeding (in months), the introduction of breast milk substitutes “formula”, the age of introducing formula, and the age of introducing complementary solid foods.

### 2.7. Additional Independent Variables (Co-Variates) 

Sociodemographic variables included the village of residence, age at enrollment, sex, household income (at baseline), and number of maternal and paternal schooling years (at follow-up assessment). Household density index at both baseline and follow-up assessment was calculated as the number of persons living in the household divided by the number of rooms in the household. Information on these variables was obtained via maternal interviews. A composite SES score was calculated using a factor analysis (expressed as Z score) and included the variables residential village, the number of maternal and paternal schooling years, household density (at follow-up assessment), and household income at baseline. Higher values of the composite SES index represent better SES.

For assessing early life growth indices, information on height and weight measurements at ages 10–14 months were obtained from medical records at the maternal and child health clinics. Weight for height Z scores (WHZ) and height for age (HAZ) Z scores at age 10–14 months were calculated using the WHO growth charts for children aged 0–59 months [32]. 

For physical activity, children where asked: “Do you regularly engage in recreational physical activity, for example: running, swimming, Karate, biking, soccer (football) or any other sport?” Replies were yes or no. 

### 2.8. Data Management 

Data obtained through interviews and measurements were subjected to quality control checks to assess completeness and consistency, and they were analyzed using Excel (Microsoft Office^®^) and IBM SPSS Version 25 (Armonk, NY, USA: IBM Corp.). WHO Anthro software and WHO Anthro Plus were used to calculate HAZ, WHZ, and BMIZ scores and compare them to the WHO growth standard population, based on information on anthropometric measurements, date of measurements, birth date and sex. The TZAMERET software and database served for entry of data from the 24 h questionnaires to generate data of the intake of various nutrients. 

### 2.9. Statistical Methods

Distributions of the study variables were assessed using histograms and Q-Q plots. Continuous variables that followed a normal distribution were described using means and standard deviations. Categorical variables were described using frequencies and percentages. 

Differences in sociodemographic and early life health-related characteristics between the study participants and children who did not participate in the study (e.g., refusals, not found) were assessed using the chi square test and Fisher’s exact test, as appropriate for categorical variables, and the Student’s *t* test for continuous variables. Spearman’s rank correlation coefficient was used to measure the correlation between early life feeding practices (the duration of breastfeeding and the age of introducing formula and solid food) and BMIZ score at school age. 

Differences in sociodemographic factors and early life exposures (e.g., gestational age at birth, birth weight, breastfeeding, and growth indices) between children with and without obesity were assessed using the chi square test for categorical variables and the Student’s *t* test for continuous variables. Dietary intake of macronutrients and fatty acids were categorized using tertiles. Differences in tertiles of macronutrient fatty acid intake between children with obesity and children without obesity were assessed using the chi square test and trend test. Logistic regression models were used to examine independent associations of dietary intake and early life exposures with obesity. The multivariable model was adjusted for daily energy intake [33]. Odds ratios (OR) and 95% confidence intervals (CI) were calculated for the independent variables, from the logistic regression model. The model fit was assessed using the Hosmer and Lemeshow test, Nagelkerke R square, and C statistics. Multicollinearity was assessed using variance inflation factors. Village of residence was stratified to explore heterogeneity of associations between the independent variables and obesity according to village. Interactions between village of residence and the independent variable were assessed using logistic regression models. A sensitivity analysis was undertaken that assessed dietary intake as a percentage of daily energy intake (categorized as tertiles). All statistical tests were 2 sided and *p* < 0.05 was considered statistically significant. 

## 3. Results

We enrolled 174 children (57% males) aged 10.3–12.4 years (mean 11.3, SD 0.5). Among the females, 25/75 (33%) had menarche. Overall, 228 (98%) children were breastfed in infancy. The proportion of children from the lower SES village was higher among those enrolled (63%) than those not enrolled in the study (40%), *p* = 0.002. No significant differences in other sociodemographic data or in early life health factors were found between children who were enrolled in the study and those who were not (Table 1).

Among participants from the lower compared to the higher SES village, mean parental schooling years and mean birth weight were higher, and mean HAZ score at age 10–14 months was lower; and a higher proportion lived in crowded households (Supplementary Table S1). 

### 3.1. Associations between Feeding Practices in Early Life and BMI at School Age

**Figure 2:**
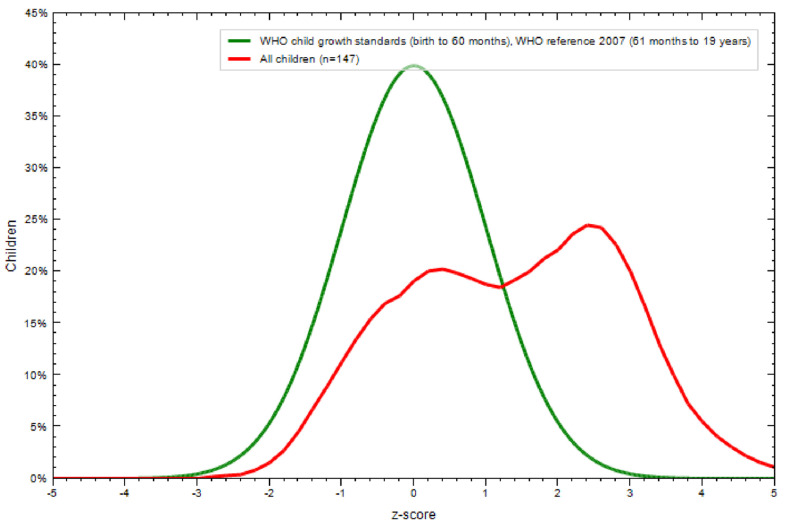
BMIZ score of the study sample compared to the 2007 WHO reference population. BMIZ of the study sample is depicted in red and that of the reference sample in green. BMIZ: Body mass index Z score; X-axis: BMIZ score; Y-axis: percentage. Two children had BMIZ scores >5 and are not represented in the figure.

The mean BMIZ score was 1.41 (SD 1.51) compared to the WHO 2007 reference population. Children of the study sample were “heavier” than those of the reference population (Figure 2).

The BMIZ score at school age was not significantly correlated with the duration of breastfeeding (Spearman’s coefficient 0.05, *p* = 0.5), the age of introducing formula (Spearman’s coefficient 0.12, *p* = 0.16), or the age of introducing solid foods (Spearman’s coefficient 0.07, *p* = 0.3).

### 3.2. Associations of Early Life Exposures and Dietary Intake at School Age with Obesity

According to the WHO classification, 65/149 (43.6%) of the children had normal BMIZ scores, 21/149 (14.1%) had overweight scores (1 ≤ BMIZ score ≤ 2SD), and 63/149 (42.3%) had obesity scores (BMIZ score > 2SD). In the following analyses, children with obesity were compared with children without obesity (i.e., children with normal weight/overweight were grouped together). Obesity was more common in those from the lower than the higher SES village (50 vs. 31%), *p* = 0.027. Children with obesity compared to those without obesity were of lower SES, but without statistical significance (*p* = 0.068). The mean gestational age at birth was significantly lower among those with than without obesity (*p* = 0.001), but mean birth weight did not differ significantly. Among children with obesity compared to children without obesity, the mean WHZ score was significantly higher at age 10–14 months (Table 2). Information on physical activity was available for 136 participants: 24/56 (42.9%) of children with obesity and 40/80 (50.0%) children without obesity reported regular recreational physical activity (*p* = 0.4).

In a bivariate unadjusted analysis, trends of higher daily dietary intake of protein and fat, especially saturated fat, were found among children with obesity compared to children without obesity (Table 3). 

No significant differences between the groups were found in daily energy intake or in dietary intake of carbohydrates, fiber, or cholesterol.

A multivariable analysis of participants of both villages, which adjusted for daily energy intake, village of residence, gestational age at birth, and WHZ score at age 10–14 months, showed a significant positive association between daily dietary intake of total fat and obesity. Compared to children at the lowest tertile of fat intake (≤46.6 g/day), those at the middle and highest tertiles of fat intake (46.7–67.8 and >67.8 g/day, respectively) were 3.8 and 5.8 times more likely to have obesity, respectively (Table 4). Replacing fat with protein, carbohydrate, fatty acids, or cholesterol yielded no significant associations between these nutrients and obesity. A positive association between WHZ at age 10–14 months and obesity at school age was found; the risk for obesity increased by 77% for each one SD increase in WHZ score. The risk for obesity decreased with higher gestational age at birth (Table 4). An additional model that included the variable composite SES score instead of residential village showed similar results (Supplementary Table S2).

### 3.3. Stratification of the Analysis by Village of Residence 

The results of the analyses stratified by village of residence are presented in the supplementary tables. No significant interactions (*p* > 0.2) were found between village of residence and associations of sociodemographic characteristics and early life exposure with obesity (Supplementary Table S3). Positive associations of protein intake and cholesterol intake with obesity (*p* = 0.013 and 0.012, respectively) were found only in the higher SES village (*p* for the interactions: 0.031 and 0.032, respectively). An interaction was found between the village of residence and intake of trans fatty acids (*p* = 0.006) (Supplementary Table S4). These results were consistent after adjustment for daily energy intake (Supplementary Table S5).

### 3.4. Sensitivity Analysis

Supplementary Table S6 presents the results of the association between dietary intake, as the percent of calories from daily energy intake, and obesity. A trend of a negative association was found between mid-tertile intake of monosaturated fat and obesity (village adjusted OR 0.42 (95% CI 0.18–0.99), *p* = 0.048). Among children from the higher SES village, a positive association was found between the highest tertile of protein intake and obesity (OR 4.74 (95% CI 0.99–22.51, *p* = 0.05)).

## 4. Discussion

The main findings of this study are the association of dietary intake of fat but not feeding practices in early life (the duration of breastfeeding and the age of introducing formula or complementary solid food) with obesity in children aged 10–12 years. Moreover, we found an association of higher WHZ score at age 10–14 months with the risk of obesity at school age. These findings are very important for the prevention of obesity in school age children, and for reducing health inequalities and cardiovascular risk in adulthood. 

Obesity was very common in the study sample, reaching 42%. While the age groups differ, this prevalence is substantially higher than that reported for Israeli Arab children in grades 7–12 in 2015–2016, in the Israel Adolescents Health and Nutrition Survey: 21.5% in boys and 12.9% in girls [24]. This discrepancy could be attributed to the lower SES of the study villages. Indeed, in the study cohort, obesity was significantly more prevalent among children from the lower than the higher SES village. These findings emphasize the need for targeted interventions for lower SES communities to reduce school age obesity and its related health consequences. Notably, the difference between the villages in obesity prevalence was not significant in a multivariable model. Interestingly, in some populations, the prevalence of obesity has been shown to be more common in children and adolescents from affluent SES strata [34,35,36]; this likely reflects greater access to high-energy dense foods. However, our findings concur with other studies [37,38], including among Jewish adolescents from Israel [39]. Accordingly, higher obesity prevalence in children from the disadvantaged than the wealthy communities might reflect lower access to and parental awareness of healthy foods. 

Understanding the impact of various macronutrient intake levels is important for preventing obesity and for formulating dietary intake recommendations for children, while considering growth and developmental needs. After adjusting for daily energy intake, residential SES, gestational age at birth, and WHZ score in infancy, the positive association between total fat intake and obesity remained significant. Compared to children at the lowest tertile of fat intake (≤46.6 g/day), those at the middle and highest tertiles were 3.8 and 5.8 times, respectively, more likely to be obese. While Recommended Dietary Allowances (RDAs) for carbohydrates and protein in children and adolescents have been determined, todate, RDAs for dietary fat intake have not been established for this age group. This is mainly due to insufficient evidence to determine a level of fat intake to prevent obesity or chronic diseases [40]. Recommendations exist for fat intake as a percent of energy intake, rather than absolute intake. Thus, our study adds new knowledge, suggesting that a daily total fat intake in the top tertile for our population might increase the risk of obesity. A prospective cohort study conducted in the Netherlands (Generation R Study) reported a positive relation between dietary protein intake at age eight years and the risk of obesity/overweigh at age 10 years [16]. However, this was mainly explained by an association of protein intake with a higher fat-free mass index [16]. Interestingly, in our sample, high protein intake and high cholesterol levels were associated with obesity only in children from the higher SES village. In both villages, trans fatty acids intake was linked positively to obesity, after adjustment for energy intake. 

Our study adds new knowledge regarding the role of early life exposures, including feeding practices in infancy and the fetal environment, in obesity at school age. We found no significant associations between breastfeeding and other early feeding practices with BMIZ scores or obesity at school age. This finding is contrary to other studies that showed a protective effect of breastfeeding against overweight/obesity, which persisted through adolescence [11], and up to age 20 years [10]. Almost all participants of our study were breastfed during infancy, as is expected in this population [27]. The near-universal breastfeeding in our cohort might explain the dissimilarity between our findings and others regarding the role of breastfeeding and obesity at school age. 

We found that higher WHZ scores at age 10–14 months increased the risk of obesity at school age, by about 77% for each one SD increase in WHZ score. This finding is in agreement with studies that showed possible associations of growth in early childhood with the risk of obesity [4,41,42,43]. Collectively, our findings and others’ emphasize the need to intervene in early life to reduce the burden of obesity and its related health consequences. 

The risk for obesity in our study decreased with increased gestational age at birth. We recruited only children born at gestational age > 34 weeks, and most participants were born as full-term babies. This precludes making inferences regarding a possible association of prematurity per se with obesity. Nonetheless, our finding adds to the body of emerging evidence regarding the importance of the fetal environment in relation to obesity and cardiometabolic health [3,4,5,6]. A population-based cohort study conducted in Israel showed higher rates of long-term pediatric endocrine and metabolic problems (including obesity) among children born at early term rather than at a later gestational age (37–38 6/7 weeks) [44]. This could be explained by differences in maturity, the need to balance the endocrine and hormonal system, or fetal endocrine dysfunction [44]. 

The two villages included in the study were of the same ethnic group and located in the same geographic region. Nonetheless, we identified differences in several correlates of obesity, including the positive association with total fat intake. This underscores the need for personalized dietary counseling. Obviously, children are dependent on their parents to provide their dietary needs, in terms of food shopping, food and drink quality and cooking practices. This emphasizes the need for dietary counseling and educational (coaching) interventions to maintain normal weight and reduce obesity among Arab children. A complete family/household approach is imperative in such interventions, given the natural child–caregiver dependence. This is especially true in the Arab traditional community, in which the mothers are typically in charge of food shopping and cooking, while the fathers are usually the main breadwinners who make major household decisions including the location for food shopping and recreational activities. 

In previous studies, we have shown that the Arab population in Israel is disproportionately affected by cardiovascular disease and diabetes mellitus as compared to the Jewish population [17], and that these disparities significantly contribute to gaps in life expectancy between the two populations [18]. The current study sheds light on early childhood origins that might affect risks of cardiometabolic diseases, including modifiable factors, such as dietary intake and obesity. Altogether, these studies highlight the need for culturally tailored lifestyle interventions, preferably starting in childhood, to reduce health disparities. 

The main limitation of our study is the refusal to participate of about 25% of the potential candidates, which resulted in a smaller sample size than we anticipated. Specifically, we expected a compliance of 85–87% based on previous studies in these villages. Our a priori power calculation did not primarily address the questions of diet/SES with obesity. Compliance of parents to participate was higher in the lower than in the higher SES village. Significant differences were not observed in other exposure variables between children whose parents consented to participate in the study and those who did not. Due to near universal breastfeeding in the study population, we could not assess differences in obesity prevalence between breastfed and non-breasted children. The 24 h recall method captures short-term dietary intake, and it might not be representative of the usual dietary intake. 

To avoid bias in dietary intake assessment, all dietary interviews were conducted during weekdays and we did not conduct interviews during school vacations or on holidays and traditional events (e.g., Ramadan, Eid) during which dietary consumption might be altered. Our study lacked information on parents’ obesity, which might be related to increased prevalence of obesity in offspring [45,46,47]. Future studies should address this question so as to better quantify risk groups for pediatric obesity. Our study has several strengths, including the prospective design, from infancy to school age; the inclusion of children from various SES backgrounds; the comprehensive assessment of early life and school age exposures; and the utilization of standardized and validated data collection tools. 

## 5. Conclusions

Feeding practices in infancy were not associated with obesity among school age children. High dietary intake of total fat at school age was associated with increased likelihood of obesity, independent of sociodemographic factors. Early life exposures such as gestational age at birth and physical growth in infancy might persistently affect the risk of obesity at school age. These findings are of high public health value for the prevention of obesity in children and reducing its related health consequences. 

## Figures and Tables

**Table 1:** Baseline characteristics compared between children who were enrolled in the follow-up study and children who were not (refusals, not found, etc.).

	Enrolled, *n* = 174	Not Enrolled, *n* = 59	*p*-Value ^a^
Village			0.002
Higher SES village (A)	64 (37%)	35 (60%)	
Lower SES village (B)	110 (63%)	24 (40%)	
Sex			0.7
Males	99 (57%)	32 (54%)	
Females	75 (43%)	27 (46%)	
Mean household crowding index (SD) in infancy ^d^	1.43 (0.69)	1.33 (0.66)	0.3
Mean maternal schooling years (SD)	10.4 (3.6)	11.1 (2.9)	0.14 ^b^
Mean paternal schooling years (SD)	10.2 (3.5)	10.6 (3.4)	0.4 ^b^
Monthly household income			0.5
≤5000 NIS	106 (62%)	32 (57%)	
>5000 NIS	66 (38%)	24 (43%)	
Duration of breastfeeding			0.2 ^c^
No breastfeeding	2 (1%)	3 (5%)	
Breastfeeding < 1 month	7 (4%)	4 (6%)	
1–3 months	47 (27%)	19 (32%)	
3.1–6 months	45 (26%)	11 (19%)	
6.1–12 months	44 (25%)	11 (19%)	
>12 months	29 (17%)	11 (19%)	
Birth weight, mean (SD), grams	3277 (445)	3194 (413)	0.2 ^b^
Gestational age at birth, mean (SD), weeks	39.1 (1.4)	39.2 (1.5)	0.6 ^b^
Age of introduction of solid food			0.3
Age ≤ 4 months	67 (39%)	27 (46%)	
Age > 4 months	107 (61%)	32 (54%)	
HAZ score at age 10–14 months, mean (SD)	−0.10 (1.10)	−0.18 (1.01)	0.6 ^b^
WHZ score at age 10–14 months, mean (SD)	0.77 (1.13)	0.81 (1.08)	0.8 ^b^

HAZ: height for age Z score; NIS: new Israeli Shekels; SD: standard deviation; SES: socioeconomic status; WHZ: weight for height Z score. *p*-value by the ^a^ Chi square test unless specified otherwise; ^b^ Student’s *t* test; ^c^ Fisher’s exact test. ^d^ Household crowding index: the number of persons living in the household divided by the number of rooms in the household. Information was missing for HAZ/WHZ at age 10–14 months (17 enrolled children; 9 not enrolled), for income (2 enrolled families; 3 not enrolled).

**Table 2:** Associations of sociodemographic and early life exposures with obesity in children aged 10–12 years.

	Obesity *n* = 63	No Obesity *n* = 86	*p*-Value ^a^
Composite SES score, mean (SD)	−0.17 (0.98)	0.15 (1.11)	0.068
Gestational age at birth, mean (SD), weeks	38.9 (1.2)	39.3 (1.4)	0.029 ^b^
Gestational age at birth			0.6
34–36 weeks	3 (5%)	3 (3%)	
37–42 weeks	60 (95%)	83 (97%)	
Birth weight, grams, mean (SD)	3292 (418)	3261 (463)	0.6
Birth weight, grams			0.8
2000–2500	3 (5%)	5 (6%)	
2501–3000	14 (23%)	20 (23%)	
3001–3500	24 (39%)	37 (43%)	
3501–4500	21 (33%)	24 (28%)	
Duration of breastfeeding, months, mean (SD)	6.6 (5.1)	6.7 (5.5)	0.9 ^b^
Breastfeeding duration			0.6
No breastfeeding	0 (0%)	2 (2%)	
Breastfeeding < 1 month	1 (2%)	5 (6%)	
1–3 months	19 (30%)	25 (29%)	
3.1–6 months	19 (30%)	19 (22%)	
6.1–12 months	16 (25%)	20 (23%)	
>12 months	8 (13%)	15 (18%)	
Age of introduction of solid food, >4 months	40 (64%)	47 (55%)	0.2
Mean WHZ score (SD), at age 10–14 months	1.09 (1.18)	0.42 (1.00)	0.001 ^b^

*p*-value was obtained by the ^a^ Chi square test unless specified otherwise; ^b^ Student’s *t* test; Data were missing for paternal schooling years (1 child with obesity) and for WHZ score (5 children with obesity and 9 without obesity). SD: standard deviation; WHZ: weight for height Z score.

**Table 3:** Differences in dietary intake of children aged 10–12 years between those with and without obesity.

	Obesity *n* = 63	No Obesity *n* = 86	*p*-Value ^a^	*p*-Trend ^b^
Energy intake, kcal			0.3	0.2
Tertile 1 (≤1462.0)	16 (25%)	32 (37%)		
Tertile 2 (1462.1–1961.6)	24 (38%)	26 (30%)		
Tertile 3 (>1961.6)	23 (37%)	28 (33%)		
Protein intake, g			0.073	0.097
Tertile 1 (≤51.6)	14 (22%)	34 (40%)		
Tertile 2 (51.7–79.9)	26 (41%)	25 (29%)		
Tertile 3 (>79.9)	23 (37%)	27 (31%)		
Carbohydrate intake, g			0.3	0.5
Tertile 1 (≤185.5)	17 (27%)	31 (36%)		
Tertile (185.6–254.2)	25 (40%)	25 (29%)		
Tertile 3 (>254.2)	21 (33%)	30 (35%)		
Fiber intake, g			0.6	0.5
Tertile 1 (≤12.98)	24 (38%)	27 (31%)		
Tertile 2 (12.99–20.3)	19 (30%)	30 (35%)		
Tertile 3 (>20.3)	20 (32%)	29 (34%)		
Total fat intake, g			0.072	0.047
Tertile 1 (≤46.6)	15 (24%)	36 (42%)		
Tertile 2 (46.6–67.8)	24 (38%)	25 (29%)		
Tertile 3 (>67.8)	24 (38%)	25 (29%)		
Cholesterol, mg			0.2	0.1
Tertile 1 (≤117.8)	16 (25%)	32 (37%)		
Tertile 2 (117.9–239.8)	23 (37%)	29 (34%)		
Tertile 3 (>239.8)	24 (38%)	25 (29%)		
Saturated fat intake, g			0.13	0.048
Tertile 1 (≤15.0)	15 (24%)	33 (38%)		
Tertile 2 (15.01–21.9)	22 (35%)	28 (33%)		
Tertile 3 (>21.9)	26 (41%)	25 (29%)		
Monounsaturated fat, g			0.2	0.1
Tertile 1 (≤16.81)	16 (25%)	33 (38%)		
Tertile 2 (16.82–24.8)	26 (41%)	28 (33%)		
Tertile 3 (>24.9)	21 (33%)	25 (29%)		
Polyunsaturated fat, g			0.6	0.4
Tertile 1 (≤6.5)	19 (30%)	32 (37%)		
Tertile 2 (6.6–15.1)	23 (37%)	28 (33%)		
Tertile 3 (>15.1)	21 (33%)	26 (30%)		

^a^ *p*-value was obtained by the chi square test and ^b^ linear-by linear association test for trends; g: gram; kcal: kilocalorie.

**Table 4:** Correlates of obesity among children aged 10–12 years.

	Unadjusted OR (95% CI)	*p*-Value ^a^	Adjusted OR (95% CI)	*p*-Value ^b^
Daily energy intake		0.6		0.6
Tertile 1 (≤1462.0)	Reference		Reference	
Tertile 2 (1462.1–1961.6)	1.85 (0.82–4.18)	0.14	0.75 (0.21–2.60)	0.6
Tertile 3 (>1961.6)	1.64 (0.73–3.71)	0.23	0.48 (0.10–2.37)	0.3
Daily total dietary fat intake		0.076		0.062
Tertile 1 (≤46.6)	Reference		Reference	
Tertile 2 (46.7–67.8)	2.30 (1.01–5.24)	0.047	3.88 (1.12–13.36)	0.032
Tertile 3 (>67.8)	2.30 (1.02–5.24)	0.047	5.84 (1.19–28.47)	0.029
Gestational age at birth, weeks	0.76 (0.59–0.98)	0.032	0.69 (0.51–0.95)	0.023
WHZ score at age 10–14 months	1.81 (1.26–2.58)	0.001	1.77 (1.21–2.59)	0.003
Village of residence				0.2
lower SES village	2.17 (1.09–4.34)	0.028	1.56 (0.68–3.56)	
higher SES village	Reference		Reference	

CI: confidence interval; OR: odds ratio; SES: socioeconomic status; WHZ: weight for height Z score. Included in the multivariable model 135 children (58 with obesity). Hosmer and Lemeshow test, *p* = 0.13. Nagelkerke R square 0.265. C statistics 0.76 (95% CI 0.68–0.84), *p* < 0.001. ^a^ *p*-value from unadjusted logistic regression model. ^b^ *p*-value from multivariable logistic regression model.

## Data Availability

Individual level data from this study cannot be made publicly available due to legal restrictions.

## Supplementary Materials

The following are available online at https://www.mdpi.com/article/10.3390/nu13062106/s1, Supplementary Table S1: Baseline characteristics of the study sample according to village of residence; Supplementary Table S2: Correlates of obesity among children aged 10–12 years—model 2; Supplementary Table S3: Associations of sociodemographic characteristics and early life exposures with obesity among children aged 10–12 years, by village; Supplementary Table S4: Associations of dietary intake with obesity among children aged 10–12 years, by village; Supplementary Table S5: Energy-adjusted associations of intakes of protein, cholesterol, and trans fatty acids with obesity, by village of residence; Supplementary Table S6: Associations of dietary intake, as percent calories from total daily energy, with obesity among children aged 10–12 years, by village.
