# Supplementary material for: Associations of Feeding Practices in Early Life and Dietary Intake at School Age with Obesity in 10- to 12-Year-Old Arab Children
Source: Nutrients. 2021 Jun 19;13(6):2106. doi: 10.3390/nu13062106 (PMC8234619; doi:10.3390/nu13062106)
Supplement: Supplementary file 1 [file nutrients-13-02106-s001.zip › nutrients-1233797-supplementary.pdf]

Supplementary table S1: Baseline characteristics of the study sample according to village of residence

	Higher SES village-A	Lower SES village-B	P value [a]
**Total**	64	110	
**Gender**			0.7
Boys	35 (55%)	64 (58%)	
Girls	29 (45%)	46 (42%)	
**Age of introduction of solid food**			0.003
Age ≤4 months	34 (53%)	33 (30%)	
Age >4 months	30 (47%)	77 (70%)	
**Breastfeeding duration**			0.2
No breastfeeding	0 (0.0%)	2 (2%)	
Breastfeeding <1 month	2 (3%)	5 (4%)	
1-3 months	17 (27%)	30 (27%)	
3.1-6 months	11 (17%)	34 (31%)	
6.1-12 months	21 (33%)	23 (21%)	
>12 months	13 (20%)	16 (15%)	
**HAZ score at age 10-14 months, mean (SD)**	0.24 (1.09)	-0.31 (1.07)	0.002 [b]
**WHZ score at age 10-14 months, mean (SD)**	0.62 (1.02)	0.86 (1.19)	0.2 [b]
**Birth weight, g, mean (SD)**	3184 (466)	3331 (422)	0.034 [b]
**Gestational age at birth, mean (SD), weeks**	39.3 (1.4)	38.9 (1.3)	0.10 [b]

P value by the [a] Chi square test unless specified otherwise; [b] Student's *t* test. [c] Household crowding index: the number of persons living in the household divided by the number of rooms in the household.
HAZ: height for age Z score; g: gram; SD: standard deviation; SES: socioeconomic status; WHZ: weight for height Z score.

Supplementary table S2: Correlates of obesity among children aged 10-12 years – model 2

	Adjusted OR (95% CI)	P value
**Daily energy intake**		
Tertile 1 (≤1462.0)	Reference	
Tertile 2 (1462.1-1961.6)	0.80 (0.22-2.84)	0.7
Tertile 3 (>1961.6)	0.52 (0.10-2.60)	0.4
**Daily total dietary fat intake**		
Tertile 1 (≤46.6)	Reference	
Tertile 2 (46.7-67.8)	3.88 (1.11-13.5)	0.034
Tertile 3 (>67.8)	5.55 (1.11-27.6)	0.036
**Gestational age at birth, weeks**	0.69 (0.50-0.94)	0.021
**WHZ score at age 10-14 months**	1.77 (1.21-2.58)	0.003
**SES composite score**	0.68 (0.38-1.21)	0.2

CI: confidence interval; OR: odds ratio; SES: socioeconomic status; WHZ: weight for height Z score. Included in the multivariable model 135 children (58 with obesity). P value from multivariable logistic regression model.

Supplementary table S3: Associations of sociodemographic characteristics and early-life exposures with obesity among children aged 10-12 years, by village

	Higher SES village-A			Lower SES village-B		
	BMIZ=<2, n=40	BMIZ>2 (obese) n=18	P value	BMIZ=<2, n=46	BMIZ>2 (obese) n=45	P value
**WHZ score at age 10-14 months, mean (SD)**	0.32 (0.84)	1.03 (1.00)	0.011	0.52 (1.13)	1.11 (1.26)	0.026
**Age of introduction of solid food**			0.2			0.9
Age ≤4 months	24 (60%)	8 (44%)		15 (33%)	15 (33%)	
Age >4 months	16 (40%)	10 (56%)		31 (67%)	30 (67%)	
**Gestational age at birth**			1.0			0.2
34-36 weeks	2 (5%)	0 (0%)		3 (7%)	1 (2%)	
37-42 weeks	38 (95%)	18 (100%)		42 (93%)	45 (98%)	
**Gestational age at birth, mean (SD), weeks**	39.6 (1.6)	39.0 (1.1)	0.19	39.2 (1.2)	38.8 (1.3)	0.15
**Birth weight, g**			0.9			0.7
2000-2500	4 (10%)	1 (5%)		1 (2%)	2 (5%)	
2501-3000	12 (30%)	7 (39%)		8 (17%)	7 (16%)	
3001-3500	13 (33%)	5 (28%)		24 (52%)	19 (43%)	
3501-4500	11 (28%)	5 (28%)		13 (29%)	16 (36%)	
**Birth weight, g, mean (SD)**	3146 (499)	3213 (389)	0.6	3361 (410)	3323 (430)	
**Breastfeeding duration**			0.9			0.4
No breastfeeding	0 (0%)	0 (0%)		2 (4%)	0 (0%)	
Breastfeeding <1 month	1 (3%)	0 (0%)		4 (9%)	1 (2%)	
1-3 months	11 (28%)	5 (28%)		14 (30%)	14 (31%)	
3.1-6 months	7 (17%)	4 (22%)		12 (27%)	15 (34%)	
6.1-12 months	13 (33%)	6 (33%)		7 (15%)	10 (22%)	
>12 months	8 (20%)	3 (167%)		7 (15%)	5 (11%)	
**Breastfeeding duration, months, mean (SD)**	7.8 (5.4)	7.7 (5.6)	0.9	5.8 (5.4)	6.2 (4.8)	0.6

BMIZ: Body mass index Z score; SD: standard deviation; SES: socioeconomic status; WHZ: weight for height Z score. P values were obtained by the chi square test. Household crowding index: the number of persons living in the household divided by the number of rooms in the household. Interactions between each of the independent variables and village of residence were assessed in separate logistic regression models that each included: village, the independent variable of interest and an interaction term between them, while obesity was the dependent variable. No significant interactions (P>0.2) were found between village of residence and any of the independent variables.

Supplementary table S4: Associations of dietary intake with obesity among children aged 10-12 years, by village

	Higher SES village-A			Lower SES village-B		
	BMIZ=<2, n=40	BMIZ>2 (obese) n=18	P value	BMIZ=<2, n=46	BMIZ>2 (obese) n=45	P value
**Energy intake , kcal**			0.10			0.9
Tertile 1 (≤1462.0)	18 (45%)	3 (17%)		14 (30%)	13 (29%)	
Tertile 2 (1462.1-1961.6)	12 (30%)	9 (50%)		14 (31%)	15 (33%)	
Tertile 3 (>1961.6)	10 (25%)	6 (33%)		18 (39%)	17 (38%)	
**Protein intake, g**			0.013			0.2
Tertile 1 (≤51.6)	21 (52%)	3 (17%)		13 (28%)	11 (25%)	
Tertile 2 (51.7-79.9)	12 (30%)	6 (33%)		13 (28%)	20 (44%)	
Tertile 3 (>79.9)	7 (18%)	9 (50%)		20 (44%)	14 (31%)	
**Total fat intake, g**			0.2			0.4
Tertile 1 (≤46.6)	20 (50%)	5 (28%)		16 (35%)	10 (22%)	
Tertile 2 (46.6-67.8)	11 (27%)	7 (39%)		14 (30%)	17 (38%)	
Tertile 3 (>67.8)	9 (23%)	6 (33%)		16 (35%)	18 (40%)	
**Carbohydrates intake, g**			0.7			0.2
Tertile 1 (≤185.5)	15 (38%)	5 (28%)		16 (35%)	12 (27%)	
Tertile (185.6-254.2)	14 (35%)	7 (39%)		11 (24%)	18 (40%)	
Tertile 3 (>254.2)	11 (27%)	6 (33%)		19 (41%)	15 (33%)	
**Fiber intake, g**			0.8			0.6
Tertile 1 (≤12.98)	15 (38%)	8 (45%)		12 (26%)	16 (36%)	
Tertile 2 (12.99-20.3)	14 (35%)	6 (33%)		16 (35%)	13 (29%)	
Tertile 3 (>20.3)	11 (27%)	4 (22%)		18 (39%)	16 (35%)	
**Cholesterol, mg**			0.012			0.5
Tertile 1 (≤117.8)	18 (45%)	3 (17%)		14 (31%)	13 (29%)	
Tertile 2 (117.9-239.8)	16 (40%)	6 (33%)		13 (28%)	17 (38%)	
Tertile 3 (>239.8)	6 (15%)	9 (50%)		19 (41%)	15 (33%)	
**Saturated fat intake, g**			0.065			0.3
Tertile 1 (≤15.0)	21 (52%)	4 (22%)		12 (26%)	11 (24%)	
Tertile 2 (15.01-21.9)	8 (20%)	8 (45%)		20 (44%)	14 (31%)	
Tertile 3 (>21.9)	11 (28%)	6 (33%)		14 (30%)	20 (45%)	
**Monounsaturated fat, g**			0.2			0.7
Tertile 1 (≤16.81)	20 (50%)	5 (28%)		13 (28%)	11 (25%)	
Tertile 2 (16.82-24.8)	11 (28%)	6 (33%)		17 (37%)	20 (44%)	
Tertile 3 (>24.9)	9 (22%)	7 (39%)		16 (35%)	14 (31%)	
**Polyunsaturated fat, g**			0.5			
Tertile 1 (≤6.5)	30 (75%)	15 (83%)		2 (4%)	4 (9%)	0.6
Tertile 2 (6.6-15.1)	8 (20%)	3 (17%)		20 (44%)	20 (44%)	
Tertile 3 (>15.1)	2 (5%)	0 (0%)		24 (52%)	21 (47%)	
**Trans fatty acids**			0.13			0.006
0	18 (45%)	6 (33%)		34 (74%)	26 (58%)	
1-2	1 (3%)	3 (17%)		7 (15%)	2 (4%)	
3+	21 (52%)	9 (5%)		5 (11%)	17 (38%)	

BMIZ: Body mass index Z score; SES: socioeconomic status. P values were obtained by the chi square test. Interactions between each of the independent variables and village of residence were assessed in separate logistic regression models that each included: total energy intake, village, each macronutrient/fatty acids of interest, and an interaction term between the variables, village and macronutrient/fatty acid, while obesity was the dependent variable. Significant interactions were found between village of residence and intake of protein (p=0.031), cholesterol (p=0.032), saturated fat (p=0.068) and trans fatty acids (p=0.006). No significant interactions (P>0.2) were found between village of residence and intake of fiber, fat, carbohydrates, monounsaturated fat or polyunsaturated fat.

Supplementary table S5: Energy-adjusted associations of intakes of protein, cholesterol, and trans fatty acids with obesity, by village of residence

	Higher SES village-A		Lower SES village-B	
	Adjusted OR (95% CI)	P value	Adjusted OR (95% CI)	P value
**Protein intake, g**		0.079		0.2
Tertile 1 (≤51.6)	Reference		Reference	
Tertile 2 (51.7-79.9)	2.98 (0.51-17.42)	0.2	1.69 (0.56-5.06)	0.3
Tertile 3 (>79.9)	8.81 (1.28-60.39)	0.027	0.64 (0.17-2.44)	0.5
**Cholesterol, mg**		0.066		0.6
Tertile 1 (≤117.8)	Reference		Reference	
Tertile 2 (117.9-239.8)	3.12 (0.43-10.48)	0.3	1.37 (0.47-3.96)	0.5
Tertile 3 (>239.8)	7.94 (1.31-47.89)	0.024	0.81 (0.27-2.41)	0.7
**Trans fatty acids**		0.058		0.009
0	Reference		Reference	
1-2	35.88 (1.68-763.82)	0.022	0.37 (0.07-1.99)	0.2
3+	0.83 (0.22-3.14)	0.78	4.76 (1.49-15.12)	0.008

CI: confidence interval; OR: odds ratio; SES: socioeconomic status. Each logistic regression model was adjusted for daily energy intake

Supplementary table S6: Associations of dietary intake, as percent calories from total daily energy, with obesity among children aged 10-12 years, by village

	Higher SES village-A			Lower SES village-B			Total		
	BMIZ=<2 n=40	BMIZ>2 (obese) n=18	P value	BMIZ=<2 n=46	BMIZ>2 (obese) n=45	P value	BMIZ=<2 n=86	BMIZ>2 (obese) n=63	P value
**Percent calories from fat**			0.5			0.8			0.4
Tertile 1	19 (47%)	7 (39%)		15 (33%)	12 (27%)		34 (40%)	19 (30%)	
Tertile 2	13 (33%)	5 (28%)		15 (33%)	16 (35%)		28 (32%)	21 (33%)	
Tertile 3	8 (20%)	6 (33%)		16 (34%)	17 (38%)		24 (28%)	23 (37%)	
**Percent calories from carbohydrates**			0.5			0.8			0.4
Tertile 1	8 (20%)	8 (44%)		16 (35%)	15 (33%)		24 (28%)	23 (37%)	
Tertile 2	12 (30%)	5 (28%)		17 (37%)	16 (36%)		29 (34%)	21 (33%)	
Tertile 3	20 (50%)	5 (28%)		13 (28%)	14 (31%)		33 (38%)	19 (30%)	
**Percent calories from protein**			0.12			0.9			0.4
Tertile 1	16 (40%)	3 (17%)		13 (28%)	17 (38%)		29 (34%)	20 (32%)	
Tertile 2	15 (38%)	7 (39%)		14 (31%)	12 (27%)		29 (34%)	19 (30%)	
Tertile 3	9 (22%)	8 (44%)		19 (41%)	16 (35%)		28 (32%)	24 (38%)	
**Percent calories from saturated fat**			0.7			0.19			0.11
Tertile 1	19 (47%)	7 (39%)		17 (37%)	9 (20%)		36 (42%)	16 (25%)	
Tertile 2	11 (28%)	5 (28%)		15 (33%)	20 (44%)		26 (30%)	25 (40%)	
Tertile 3	10 (25%)	6 (33%)		14 (30%)	16 (36%)		24 (28%)	22 (35%)	
**Percent calories from monounsaturated fat**			0.07			0.4			0.08
Tertile 1	13 (33%)	7 (39%)		13 (28%)	17 (38%)		26 (30%)	24 (38%)	
Tertile 2	16 (40%)	2 (11%)		18 (39%)	12 (27%)		34 (40%)	14 (22%)	
Tertile 3	11 (27%)	9 (50%)		15 (33%)	16 (35%)		26 (30%)	25 (40%)	
**Percent calories from polyunsaturated fat**			0.4			0.8			0.3
Tertile 1	31 (77%)	16 (89%)		2 (4%)	1 (2%)		33 (38%)	17 (27%)	
Tertile 2	7 (18%)	2 (11%)		21 (46%)	22 (49%)		28 (33%)	24 (38%)	
Tertile 3	2 (5%)	0 (0%)		23 (50%)	22 (49%)		25 (29%)	22 (35%)	

BMIZ: Body mass index Z score; SES: socioeconomic status. P value was obtained by the chi square test.
